# Mortality in the Medical Profession

**Published:** 1887-02

**Authors:** 


MORTALITY IN THE MEDICAL PROFESSION.

The long list of deaths which occurred in the ranks of the medical profession during the year 1886, both at home and abroad, embraces the names of many who have, for years, been recognized as leading spirits in the profession, but who are now numbered among those who have passed away.

In this country no medical man of his generation has occupied a more prominent position in the profession than Professor Austin Flint, M. D.,LL.D.

At the time of his death he was little less than seventy-four years of age, and he was as rich in honors as he was in years.

By his works as well as personally, he was well known and esteemed on both sides of the Atlantic ocean.

In addition to the official positions which he occupied in this country, he was the President-elect of the Ninth International Medical Congress, which is to meet in Washington, D. C., in September of this year. Whilst in the death of Professor Flint general medicine lost one of the brightest lights in this country, so general surgery lost, in the death of Professor Alfred C. Post, M.D., and Frank H. Hamilton, M.D., two of its representative men. Both were rich in years and in good work, faithfully performed.

Among the names of other eminent men who died during the year should be embraced the names of Professors John P.

Gray, M.D.,LL.D., of Utica, J. F. Miner of Buffalo, W. H. Dudley of Brooklyn, J. G. Richardson of Philadelphia.

In England, two of the most prominent deaths during the year were Drs. Walter Moxon of Guy's Hospital, and James G. Wakley of the London *Lancet*.

In France, Professor Paul Bert, the eminent physiologist, Dr. Le Grand Du Saulle, of La Salpêtriere, Dr. Brochfontaine of the Laboratory of the Faculty of Medicine of Paris, Dr. Arvedec De Chambre, editor of the *Gazette Hebdomodaire*, of Paris, are among the notable deaths.

In other continental countries of Europe, death has claimed among its victims Professor Theodore Friederick Von Frerichs of Berlin, Professor Von Gudden, whose lamentable death with King Ludwig of Bavaria is well remembered; Professor Auspitz, editor of the *Archives für Dermatologie und Syphilis*; Professor Hermann Maas of Würzburg, Professor Grobe of Greifswald, Professor Lushinger of Zurich, Professor A. Burckhardt-Merian of Basle, Professor C. G. Santesson of Stockholm, and, not the least, Professor Panum of Copenhagen.

The works of these great men are so numerous and so well known as need no mention here, even if space permitted but a partial recapitulation. Most of them are monuments to their memory, which are enduring, even after their authors have passed away.

